# Genotypes of hepatitis a virus in Turkey: first report and clinical profile of children infected with sub-genotypes IA and IIIA

**DOI:** 10.1186/s12879-017-2667-3

**Published:** 2017-08-11

**Authors:** Huseyin Yilmaz, Asiye Karakullukcu, Nuri Turan, Utku Y. Cizmecigil, Aysun Yilmaz, Ayse A. Ozkul, Ozge Aydin, Alper Gunduz, Mahmut Mete, Fadile Y. Zeyrek, Taner T. Kirazoglu, Juergen A. Richt, Bekir Kocazeybek

**Affiliations:** 1 Department of Virology, Veterinary Faculty, University of Istanbul, Avcilar, Istanbul, Turkey; 2 Department of Microbiology, Cerrahpasa Faculty of Medicine, University of Istanbul, Cerrahpasa, Istanbul, Turkey; 3 Department of Child Health and Diseases, Faculty of Medicine, University of Izmir, Karsiyaka, Izmir Turkey; 4 Division of Infectious Diseases and Clinical Microbiology, Sisli Hamidiye Etfal Training and Research Hospital, Istanbul, Turkey; 5 Department of Microbiology, Faculty of Medicine, University of Dicle, Diyarbakir, Turkey; 6 Department of Microbiology, Faculty of Medicine, University of Harran, Urfa, Turkey; 7 Department of Diagnostic Medicine and Pathobiology, College of Veterinary Medicine, Kansas State University, Manhattan, USA

**Keywords:** Hepatitis a virus sequencing, Phylogenetic, VP1/VP2A, VP1/VP3, Turkey

## Abstract

**Background:**

Hepatitis A virus (HAV) is a food and water-borne virus causing clinical (mainly hepatitis) and subclinical disease in humans. It is important to characterize circulating strains of HAV in order to prevent HAV infections using efficacious vaccines. The aim of this study was the detection and characterization of the circulating strains of HAV in Turkey by performing serology, RT-PCR, sequencing and phylogenetic analysis.

**Methods:**

In this study, 355 HAV suspected cases were analysed by ELISA for the presence of antibodies to HAV. RNA was extracted from 54 HAV IgM positive human sera. None of the suspect cases were vaccinated against HAV and they never received blood transfusions. Samples found positive by RT-PCR using primers targeting the VP1/VP2A junction and VP1/VP3 capsid region of HAV, were subjected to sequencing and phylogenetic analyses.

**Results:**

IgM type antibodies to HAV were detected in 54 patients. Twenty one of them were students. The age of IgM positive cases was between 3 and 60 years. IgM positivity differed in age groups and was higher in the age group 3 to 10 years. Phylogenetic analysis showed that the majority of HAV strains detected in this study belong to the “HAV 1B” cluster. In addition, the HAV sub-genotypes IA (KT874461.1) and IIIA (KT222963.1) were found in 2 children. These sub-genotypes were not previously reported in Turkey. The child who carried sub-genotype IIIA travelled to Afghanistan and presented with abdominal pain, icterus and vomitus. He was positive for anti-HAV IgM and IgG but negative for hepatitis B and C. Liver enzymes like aspartate aminotransferase, alanine aminotransferase, alkaline phosphatase, gamma-glutamyl transferase and lactate dehydrogenase were severely elevated. Bilirubin levels were also increased. White blood cells, neutrophils and hemoglobin were decreased while lymphocytes and monocytes were increased. Similar clinical signs and laboratory findings were reported for the child infected with sub-genotype IA but aspartate aminotransferase and alanine aminotransferase were not severely elevated.

**Conclusions:**

The results indicate that molecular studies determining the HAV genotype variation in Turkey are timely and warranted. The majority of IgM positive cases in 3–10 year old patients indicate that childhood vaccination is important. Sub-genotype IB is the most prevalant genotype in Turkey. Surprisingly, sub-genotype IA and IIIA are also present in Turkey; future diagnostic efforts need to include diagnostic methods which can identify this emerging HAV genotypes. Our results also show that one important risk factor for contracting hepatitis A virus is international travel since genotype IIIA was detected in a child who had travelled to Afghanistan.

## Background

Hepatitis A virus (HAV) infections occur worldwide with approximately 1.4 million new cases reported each year with 11–22% of the cases being hospitalized [1]. The severity and symptoms of HAV infections are strongly associated with age. HAV infections are asymptomatic in childhood while symptoms like jaundice and acute liver failure are more prominent in older people with mortality rates up to 1.8%, depending on the region and vaccination policy [2, 3].

Hepatitis A virus is a non-enveloped RNA virus with positive polarity and the only member of genus *Hepatovirus* in the family *Picornaviridae*. The viral genome is about 7.5 kb and contains 5′ and 3′ untranslated regions and one open reading frame (ORF) [4]. The open reading frame of HAV RNA encodes a large polyprotein (P0) with 2227 amino acid residues. Protease cleavage of the HAV polyprotein yields four major proteins which are VP1- VP4. It has 3 regions as P1, P2 and P3. The P1 region encodes capsid or structural proteins, 1A (VP4), 1B (VP2), 1C (VP3) and 1D (VP1). The P2 and P3 regions encode non-structural proteins, 2A, 2B and 2C, and 3A, 3B, 3C and 3D, respectively [5–7].

Complete genome sequence analysis have shown that HAV can be divided into 7 genotypes [7, 8]. Genotypes I, II, III and VII have been associated with hepatitis in humans. Most of the HAV strains detected in humans belong to genotypes I and III with 80% of them being genotype I. Genotypes I and III are divided in to sub-genotypes A, B and C. Genotypes IV, V and VI are found in simians and have been also recovered from pigs [7, 8].

HAV is transmitted by the feco-oral route mainly through contaminated food (sandwiches, fruits, salads and shellfish) and water and to a lesser extend from person to person or via blood transfussion [9–12]. Hepatitis A virus is endemic mainly in the Middle East, Asia and in North African countries [3] but outbreaks have also been reported in Europe [13, 14], especially in tourists returning from HAV endemic countries. Incidence rates of HAV are strongly associated with socioeconomic factors correlating with poor hygienic conditions, which affects the hygiene of food and water [2]. However, with improvement of food safety, HAV incidence in endemic countries decreased in young children but increased in adolescents and adults [3]. Since mainly children are vaccinated in many countries, elderly people are more affected by HAV in recent years because of the decrease in vaccine immunity to HAV.

In order to prevent HAV infections, it is important to know the HAV strains circulating in an endemic area in order to establish effective vaccination strategies. Several regions of the HAV genome have been used for genotyping to investigate molecular variants of HAV. These are the entire VP1 region, the N terminus of the VP1 region, the VP1–2A junction region, the VP1–2B region, the VP3-2B region, the C terminus of the VP3 region and the 5’UTR region [7, 15–18]. The aim of this study was detection and investigation of the circulating strains of HAV in Turkey by performing serology, RT-PCR, sequencing and phylogenetic analysis.

## Methods

### Patients and diagnosis of hepatitis A

The study population consisted of 355 patients who showed one or two clinical symptoms of hepatitis (abdominal pain, diarrhea, vomiting and/or icterus) and were admitted to the clinical department at the Cerrahpasa Medical School Hospital in Istanbul, Turkey, between 2012 and 2015 as well as the Sisli Research Hospital, Dicle University, Izmir University and Harran University. The clinical symptoms, age, gender, occupation, travel history and vaccination status were recorded. Sera were collected for serological and molecular analyses in the first week after the onset of clinical symptoms. Samples were transported to the research laboratory using cold storage (4°–8 °C). In order to confirm hepatitis A diagnosis, all sera were analysed by an ELISA for the presence of IgM antibodies to HAV as described in detail by the manufacturer (HEPAVASE MA-96, TMB; General Biologicals Corporation, Germany).

### RNA extraction and cDNA synthesis

For the extraction of viral RNA, QIAamp Viral RNA mini kit (Qiagen, Germany) was used to extract RNA from 54 sera which were found to be IgM positive to HAV. Extraction procedures were carried out according to the instructions described by the manufacturer (Qiagen, Germany). The amount of RNA in the extracted material was measured using a NanoDrop spectrophotometer (NanoDrop 1000c, Thermo Scientific). Approximately 500–600 ng/μl RNA was used for reverse transcription (RT). RT was performed in 2 steps. For the first step, 9 μl of RNA template (about 400 ng) was mixed with 1 μl random hexamers (Promega) and incubated at 70 °C for 5 min, followed by a cooling step to 4 °C using a thermal cycler (Biorad Chromo4). For the second step, a total volume of 20 μl reaction mixture was prepared consisting of 10 μl RNA/primer mixture from the first step, 4 μl 5X RT buffer, 2.4 μl 25 mM MgCl_2_, 1 μl 10 mM dNTPs, 1.6 μl nuclease-free water, and 1 μl reverse transcriptase (Improm II, Promega). The mixture was returned to the thermal cycler and incubated at 20 °C for 5 min, 42 °C for 30 min and 70 °C for 15 min before being cooled to 4 °C. After completion of the RT reaction, 30 μl of nuclease-free water was added to each cDNA sample and the samples were kept at −80 °C until required.

### Sequencing and phylogenetic analyses

Samples (*n* = 54) found positive by IgM ELISA were subjected to RT-PCR, sequencing and phylogenetic analyses. Commercially synthesized primers were used in these analyses (DNA Technology, Denmark). Two different PCR reactions were set up using primers targeting the VP1/VP2A junction region and the VP1/VP3 region (Table 1). The primers and method used to amplify the VP1/VP3 region were the same as described previously [16]. However, in order to amplify the VP1/VP2A junction region, a modification of the previous method was used [15]. For this purpose, a nested PCR was performed. For the first amplification step, the newly designed primers (HAV-OtF and HAV-Ot-R) were used (Table 1). Briefly, a total volume of 25 μl reaction mixture was prepared for an optimized PCR consisting of 12.5 μl Maxima Hot Start PCR Master Mix (Thermo Scientific), 1 μl HAV-OtF primer (2.5 pmol/μl), 1 μl HAV-OtR primer (2.5 pmol/μl), 0.5 μl MgCl_2_ (25 mM), 5 μl nuclease free water and 5 μl cDNA. The mixture was placed in a thermal cycler (Stratagene mx3000p). Cycling conditions were as follows: 95 °C for 4 min then 40 cycles of 94 °C for 40 s, 54 °C for 1 min, 72 °C for 1 min and final incubation at 72 °C for 7 min. For the second nested amplification step, primers (HAV BR5F and HAV newRT) (Table 1) and a method described by Reuter and others (2006) were used. Briefly, a total volume of 50 μl reaction mixture was prepared for an optimized PCR consisting of 25 μl Maxima Hot Start PCR Master Mix (Thermo Scientific), 1 μl BR5F primer (20 pmol/μl), 1 μl newRT primer (20 pmol/μl), 21 μl nuclease free water and 2 μl first step product. The mixture was placed in a thermal cycler (Stratagene mx3000p). Cycling conditions were as follows: 95 °C for 4 min then 40 cycles of 94 °C for 1 min, 58 °C for 1.5 min, 72 °C for 1 min and final incubation at 72 °C for 7 min. After PCR, a HAV-specific 360 bp product was visualized by 1.5% agarose gel electrophoresis.

**Table 1:** Primers and their sequences used in this study

Primers	Sequences 5’-3’	Product size	Target gene	Target Sequences	References
HAV-ot-VP1–2- 2848 F	GAGCACTGGATGGTTTGGGW	466 bp	VP1/VP2A junction	2848–2867	Designed in this study
HAV-ot-VP1–2- 3314 R	WGCAGTCACWCCTCTCCAAG			3314-3295^a^	
HAV BR5F	TTGTCTGTCACAGAACAATCA	360 bp	VP1/VP2A junction	2952–2973	Reuter et al., 2006
HAV newRT	AGC AGT CAC TCC TCT CCA G			3294–3311^b^	
HA2F	GTTTTGCTCCTCTTTATCATGCTATG	247 bp	VP1-VP3 capsid	2165–2191	Namsai et al., 2011
HA1R	GGAAATGTCTCAGGTACTTTCTTTG			2411–2387^c^	

^a^M20273; ^b^M14707 and ^c^KP879217 indicates the sequences derived from the GenBank accession numbers

For all RT-PCR reactions, nuclease free water was used as negative control in place of a template. Positive controls were obtained from sera samples submitted to Cerrahpasa Hospital laboratory, which were previously confirmed to be HAV positive by RT-PCR and sequencing. Products obtained by PCR using the primers specific for the VP1/VP2A and VP1/VP3 regions were sequenced by a commercial company (MedSanTek, Istanbul, Turkey; REFGEN, Ankara, Turkey). Multiple alignments of VP1/VP2A and VP1/VP3 region sequences were made using the Mega 7 software. Phylogenetic analyses were carried out using the criterion of neighbour-joining trees based on genetic distance model by Tamura-Nei [19]. After the generation of phylogenetic trees, the sequences were analyzed for the presence of possible recombination event or recombinant HAV strains.

## Results

### Clinical findings and demography of HAV IgM positive cases

HAV-specific IgM antibodies were detected in 54 patients. All patients with IgM type antibodies to HAV were suffering from one or two of the following clinical signs: abdominal pain, diarrhea, vomitus and/or icterus. None of these patients were vaccinated against HAV or received a blood transfusion. Twenty one (*n* = 21) of them were students.. Fourty three (*n* = 43) cases were from Istanbul, 8 cases from the east of Turkey and 3 cases from the west of Turkey. Two (*n* = 2) patients have recently travelled to Afghanistan. Thirty four (*n* = 34) of them were male and 20 were female. The ages of IgM positive cases were between 3 and 60 years old. IgM positivity differed in age groups and was higher in ages between 3 to10 years (Table 2). Various characteristics of the HAV IgM positive patients and some factors like age, occupation, eating habit, sea food consumption and presence of icterus in patients are summarized in Table 2.

**Table 2:** Characteristics and some factors associated with HAV IgM positive patients by age groups

	Age Groups (Year)					
	3–10	11–20	21–30	31–40	41–50	51–60
Sex (%)						
Male	15 (68)	6 (50)	9 (75)	2 (66.6)	2 (66.6)	0 (0)
Female	7 (32)	6 (50)	3 (25)	1 (33.4)	1 (33.4)	2 (100)
Job (%)						
Student	12 (54.5)	12 (100)	3 (25)	0 (0)	0 (0)	0 (0)
Waiter	0 (0)	0 (0)	1 (8.3)	0 (0)	0 (0)	0 (0)
Sailor	0 (0)	0 (0)	1 (8.3)	0 (0)	0 (0)	0 (0)
Others	0 (0)	0 (0)	1 (8.3)	3 (100)	3 (100)	1 (50)
Non-working	10 (45.5)	0 (0)	6 (50)	0 (0)	0 (0)	1 (50)
City of residence (%)						
Istanbul	14 (63.6)	11 (91.6)	10 (83.3)	3 (100)	3 (100)	2 (100)
Other cities	8 (36.4)	1(8.4)	2 (16.7)	0 (0)	0 (0)	0 (0)
Eating habit (%)						
Homemade	19 (86.3)	4 (33.3)	9 (75)	3 (100)	3 (100)	2 (100)
Fast food	3 (13.7)	8 (66.7)	3 (25)	0 (0)	0 (0)	0 (0)
Consumption of seafood (%)						
Fish	1 (4.5)	0 (0)	3 (25)	0 (0)	1 (33.4)	1 (50)
Mussels	1 (4.5)	1(8.4)	0 (0)	0 (0)	0 (0)	0 (0)
No seafood	20 (91)	11 (91.6)	9 (75)	3 (100)	2 (66.6)	1 (50)
Presence of symptoms (%)						
Icterus	9 (41)	7 (58.3)	1 (8.4)	1 (33.4)	2 (66.6)	1 (50)
Others^a^	13 (59)	5 (41.7)	11 (91.6)	2 (66.6)	1 (33.4)	1 (50)

None of the patients received blood transfusion. ^a^Presence of vomitus and/or abdominal pain and/or diarrhea

### Sequencing and phylogenetic analyses

#### VP1/VP2A region

**Fig. 1:**
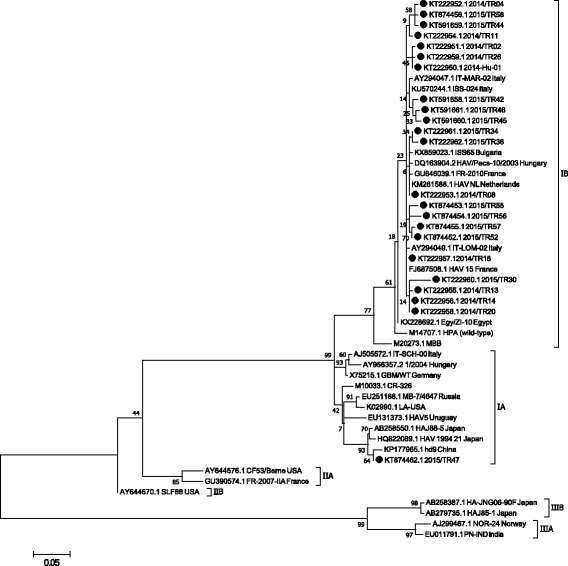
Phylogenetic tree generated by using the sequences obtained from the VP1/VP2A junction. Phylogenetic tree, using Neighbour Joining and Bootstrap analysis, was generated following alignment of the sequences, by Mega 7, and compared with partial VP1/VP2A junction sequences of HAV strains deposited in GenBank from other investigators in Turkey and other countries

Amplification of a 360 bp product of the VP1/VP2A region of HAV was successful with 23 out of 54 IgM positive sera. The partial VP1/VP2A sequences detected in 22 sera (Fig. 1) were closely related to HAV sub-genotype IB strains detected in Netherlands (KM261588.1), Hungary (DQ163904.2), France (GU646039.1), Italy (AY294047), Bulgaria (KX859023.1) and Egypt (KX228692.1). In addition, HAV sub-genotype IA (KT874462.1) was detected in one serum and was closely related to HAV sub-genotype IA strains previously reported in China (KP177965.1) and Japan (AB258550.1), but slightly different than those reported in United States of America (K02990.1), Russia (EU251188.1), Germany (X75215.1) and Italy (AJ505572.1) (Fig. 1). No recombination or circulating recombinant forms were present amongst the strains detected when analyzing the HAV VP1/VP2 junction region.

#### VP1/VP3 region

**Fig. 2:**
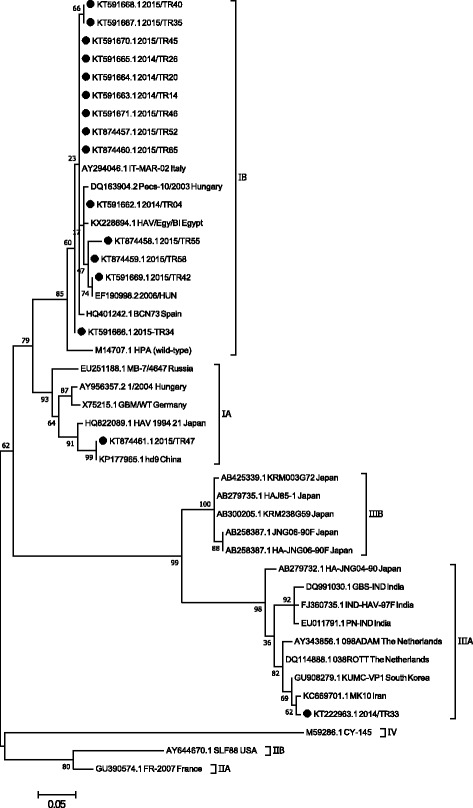
Phylogenetic tree generated by using the sequences obtained from the VP1/VP3 capsid region. Phylogenetic tree, using Neighbour Joining and Bootstrap analysis, was generated following alignment of the sequences, by Mega 7, and compared with partial VP1/VP3 capsid sequences of HAV strains deposited in GenBank from other investigators in Turkey and other countries

Amplification of a 247 bp product of the VP1/VP3 region of HAV was successful with 16 out of 54 IgM positive sera. The partial VP1/VP3 sequences detected in 14 sera (Fig. 2) were similar to HAV sub-genotype IB strains detected in Hungary (EF190998.2), Spain (HQ401242.1), Italy (AY294046.1), Egypt (KX228694.1) but different from HAV genotypes IA, IIA, IIB, IIIA and IIIB (Fig. 2). In addition, HAV sub-genotype IA was found in one serum and sub-genotype IIIA in another serum; these 2 sub-genotypes were not previously detected in Turkey. The HAV sub-genotype IA (KT874461.1) detected in this study is closely related to IA strains previously reported in China (KP177965.1) and Japan (HQ822089.1) but slightly different than those reported in Hungary (AY956357.2), Russia (EU251188.1), and Germany (X75215.1) (Fig. 2). The HAV sub-genotype IIIA (KT222963.1) detected in this study is closely related to HAV sub-genotype IIIA strains previously reported in Iran (KC669701), Netherlands (AY343856.1) and South Korea (GU908279.1) but slightly different than those reported in Japan (AB279732.1) and India (DQ991030.1). No recombination or circulating recombinant forms were present amongst the strains detected when analyzing the HAV VP1/VP3 region.

### Clinical signs and laboratory findings of children infected with HAV sub-genotype IA and IIIA

The child which was found positive for HAV sub-genotype IIIA (KT222963.1) had abdominal pain, icterus, vomitus and a travel history to Afghanistan. He was positive for anti-HAV IgM and IgG but negative for hepatitis B and C. He had elevated levels of total IgM, IgG and IgE. Liver enzymes like aspartate aminotransferase, alanine aminotransferase, alkaline phosphatase, gamma-glutamyl transferase and lactate dehydrogenase were very high. Bilirubin levels were also increased. White blood cells, neutrophils and hemoglobin were decreased while lymphocytes and monocytes were increased (Table 3).

**Table 3:** Serological, biochemical and heamatological test results of the child positive for HAVsub-genotype IIIA

Parameters	Results	Units	Reference Values
Total IgM	914,4^a^	mg/dl	40–320
Total IgA	153,9	mg/dl	34–305
Total IgG	1852^a^	mg/dl	700–1600
Total IgE	292^a^	IU/ml	<100
Anti-HBs	33	IU/ml	-
HBs Ag	Negative	-	Negative
Anti-HCV	Negative	-	Negative
Anti-HAV IgM	Positive	-	Negative
Anti-HAV IgM	Positive	-	Negative
AST	2555^a^	IU/L	0–40
ALT	2163^a^	IU/L	0–40
ALP	252^a^	IU/L	20–155
GGT	165^a^	IU/L	3–25
LDH	1066^a^	IU/L	<764
Total protein	5,6^a^	g/dl	6–8
Albumin	2,9^a^	gr/dl	3,2–5,4
CRP	0,5	mg/dl	0–0,5
Urea	8^a^	mg/dl	10–50
Uric acid	2,2	mg/dl	2–5,5
Creatinine	0,3	mg/dl	0,3–1,1
Total bilirubin	3,26^a^	mg/dl	0,3–1,2
Direct bilirubin	3,09^a^	mg/dl	0–0,2
WBC	4,1^a^	10^3^mm^3^	5,2–12,4
RBC	4,3	10^6^mm^3^	4–6,2
HGB	10,9^a^	g/dl	12–18
HCT	33^a^	%	37–52
PLT	574^a^	10^3^mm^3^	130–400
NEUT %	27^a^	%	40–74
LYMPH %	58,3^a^	%	19–48
MONO %	17,8^a^	%	3,4–9
EOS %	1,8	%	0–7
BASO %	0,3	%	0–1,5

*AST* aspartate aminotransferase, *ALT* alanine aminotransferase, *ALP* alkaline phosphatase, *GGT* gamma-glutamyl transferase, *LDH* lactate Dehydrogenase, *CRP* c-reactive protein, *WBC* white blood cell, *RBC* red blood cell, *HGB* hemoglobin, *NEUT* neutrophil, *LYMPH* lymphocyte, *MONO* monocyte, *EOS* eosinophil, *BASO* basophil; ^a^Values below or above the reference range

Similar clinical signs and laboratory findings were reported for the child infected with sub-genotype IA, but liver enzymes aspartate aminotransferase and alanine aminotransferase were not very high.

## Discussion

Hepatitis A virus is an important human pathogen, reported worldwide including Turkey. Undercooked seafood, vegetables, fruits, ready-to –eat food and water are the main source of infection [9, 11, 20]. In order to prevent HAV infections ms, it is important to know the virus source, circulating genotypes and variants of HAV by performing molecular epidemiology. In many studies, the VP1–2A junction of HAV was choosen for analysis since this junction is the most variable region of the HAV genome [14, 18]. Results of previous studies have shown that genotype I is more prevalent in Europe and America than in other continents while genotype III is endemic in Asia [21–24]. However, genotype III has recently been reported in Spain [18]. Sub-genotype IA has been circulating mainly in Mediterranean countries like Greece, Italy and France whereas sub-genotype IB seems to be present in Spain, Jordan and Egypt [3, 18, 22]. Similar to previous studies from Turkey [17, 25]^,^ sub-genotype IB was detected in the majority of patients when the VP1/VP2A junction and VP1/VP3 capsid region of HAV was amplified.

Sequencing and phylogenetic analyses of this study targeting the VP1/VP2A junction region revealed that 22 out of 23 HAVs clustered as sub-genotype IB. The IB sub-genotype was confirmed with 14 of those sera when the VP1/VP3 region was analyzed. The Turkish IB sub-genotype is similar to the one reported in Netherlands, Hungary, France, Italy, Bulgaria and Egypt (Fig. 1). In addition, sub-genotype IA and IIIA strains were found for the first time in Turkey; both sub-genotypes were detected in sera of children. Sub-genotype IIIA was detected by using the primers targeting the VP1/VP3 region but not the VP1/VP2A junction region (Fig. 2). This indicates the importance of targeting different regions of the HAV genome to detect different genotypes. Interestingly, the travel history of the child carrying sub-genotype IIIA indicated that the child had travelled to Afhganistan. The sub-genotype IIIA (KT222963.1) detected in this study, associated with rather severe clinical symptoms, was found to be similar to strains found in Iran, The Netherlands, South Korea, Japan and India (Fig. 2). These data indicate that the Turkish HAV sub-genotype IIIA detected might have originated from Iran or India, and made his way through Afghanistan to Turkey (Barde et al., 2014). The sub-genotype IA detected in this study was found to be similar to HAV sub-genotype IA reported in China, Japan, Russia, Hungary and Germany (Figs. 1 and 2). So far, these newly detected genotype IA and IIIA were only found in 2 children, but more epidemiological studies are needed to understand the real distribution of HAV sub-genotypes IA and IIIA in Turkey. If these novel sub-genotypes are more widely spread, a new vaccination policy might need to be considered since these sub-genotypes seem to be causing severe disease in children [18, 26].

As it was reported previously, Turkey is considered to be an intermediate endemic region for HAV [17, 25]. In this study, age distribution of HAV positives was different with a higher incidence in children than in adults. This confirms the results reported in another studies [17, 25] performed in Turkey, indicating that childhood vaccination program should continue particularly in endemic areas in Turkey. In fact, until September 2012, a hepatitis A vaccine containing genotypes (A HM175, CR 326, GBM starin) was used in children as a voluntary immunization (Ministry of Health, Turkey). After September 2012, children are being routinely vaccinated at 18 and 24 months old age in Turkey (Ministry of Health, Turkey). These vaccination regime seems to provide lifelong protection against the currently circulating HAV strain sub-genotype IB [27]. Howerver, further investigations are necessary to evaluate the current vaccines in protection against the newly identifed HAV genotypes IA and IIIA in Turkey.

## Conclusion

The present study with the identification of novel HAV-subgenotypes indicates that molecular studies determining the HAV genotype variation in Turkey are warranted. The majority of IgM positive cases in 3–10 year old patients indicates that childhood vaccination in Turkey is important. Sub-genotype IB is the most prevalent genotype in Turkey. Surprisingly, sub-genotype IA and IIIA are also present in Turkey; therefore, future diagnostic efforts should include methods which can identify these emerging HAV genotypes. Our results also show that one important risk factor for contracting hepatitis A virus is travelling since genotype IIIA was detected in a child who had travelled to Afghanistan.

## Abbreviations

cDNA: Complementary DNA
HAV: Hepatitis A virus
IgM: Immunoglobulin M
RT-PCR: Reverse transcriptase PCR
VP: Viral protein
